# Inhibitory effects of endomorphin-2 on excitatory synaptic transmission and the neuronal excitability of sacral parasympathetic preganglionic neurons in young rats

**DOI:** 10.3389/fncel.2015.00206

**Published:** 2015-05-28

**Authors:** Ying-Biao Chen, Fen-Sheng Huang, Ban Fen, Jun-Bin Yin, Wei Wang, Yun-Qing Li

**Affiliations:** ^1^Department of Anatomy, Histology and Embryology, Fujian Medical UniversityFuzhou, China; ^2^Department of Anatomy, Histology and Embryology and K. K. Leung Brain Research Centre, The Fourth Military Medical UniversityXi’an, China; ^3^Division of Medical Biophysics, Institute of Neuroscience and Physiology, Göteborg UniversityGöteborg, Sweden; ^4^Collaborative Innovation Center for Brain Science, Fudan UniversityShanghai, China

**Keywords:** endomorphin-2, parasympathetic preganglionic neurons, sacral parasympathetic nucleus, neuronal excitability, spontaneous excitatory postsynaptic current, urinary retention

## Abstract

The function of the urinary bladder is partly controlled by parasympathetic preganglionic neurons (PPNs) of the sacral parasympathetic nucleus (SPN). Our recent work demonstrated that endomorphin-2 (EM-2)-immunoreactive (IR) terminals form synapses with μ-opioid receptor (MOR)-expressing PPNs in the rat SPN. Here, we examined the effects of EM-2 on excitatory synaptic transmission and the neuronal excitability of the PPNs in young rats (24–30 days old) using a whole-cell patch-clamp approach. PPNs were identified by retrograde labeling with the fluorescent tracer tetramethylrhodamine-dextran (TMR). EM-2 (3 μM) markedly decreased both the amplitude and the frequency of the spontaneous and miniature excitatory postsynaptic currents (sEPSCs and mEPSCs) of PPNs. EM-2 not only decreased the resting membrane potentials (RMPs) in 61.1% of the examined PPNs with half-maximal response at the concentration of 0.282 μM, but also increased the rheobase current and reduced the repetitive action potential firing of PPNs. Analysis of the current–voltage relationship revealed that the EM-2-induced current was reversed at −95 ± 2.5 mV and was suppressed by perfusion of the potassium channel blockers 4-aminopyridine (4-AP) or BaCl_2_ or by the addition of guanosine 5′-[β-thio]diphosphate trilithium salt (GDP-β-S) to the pipette solution, suggesting the involvement of the G-protein-coupled inwardly rectifying potassium (GIRK) channel. The above EM-2-invoked inhibitory effects were abolished by the MOR selective antagonist D-Phe-Cys-Tyr-D-Trp-Orn-Thr-Pen-Thr-NH2 (CTOP), indicating that the effects of EM-2 on PPNs were mediated by MOR via pre- and/or post-synaptic mechanisms. EM-2 activated pre- and post-synaptic MORs, inhibiting excitatory neurotransmitter release from the presynaptic terminals and decreasing the excitability of PPNs due to hyperpolarization of their membrane potentials, respectively. These inhibitory effects of EM-2 on PPNs at the spinal cord level may explain the mechanism of action of morphine treatment and morphine-induced bladder dysfunction in the clinic.

## Introduction

Morphine is an exogenous ligand of the μ-opioid receptor (MOR) and is the only FDA-approved opioid for intrathecal administration. Morphine is effective, inexpensive, and well tolerated by the majority of patients (Winkelmüller and Winkelmüller, 1996; Ruan, 2007). However, the clinically relevant side effects of the intrathecal administration of morphine, including urinary retention, have largely limited its clinical application (Ruan, 2007). Although morphine can inhibit bladder activity when it is administered into cervical, thoracic or lumbar regions, this inhibitory effect is faster acting and more effective when it is administered directly in the vicinity of the lumbosacral spinal cord (Dray and Metsch, 1984a). Additionally, urinary retention has not been reported following intraventricular morphine administration (Ruan, 2007). Moreover, the detrusor relaxation caused by epidural morphine in humans is readily reversed with naloxone (Rawal et al., 1983). All of these results indicate that morphine, the exogenous ligand of MOR, is involved in the neurogenic inhibition of bladder motility via spinal mechanisms (Dray and Metsch, 1984a,b).

Previous studies have revealed that the sacral parasympathetic nucleus (SPN), which is located in the intermediolateral region of the caudal lumbar and rostral sacral (L6-S1) spinal segments in the rat (Nadelhaft and Booth, 1984; Nadelhaft and Vera, 1995; Miura et al., 2000, 2001, 2003; Vera and Nadelhaft, 2000; Dou et al., 2013), is a spinal autonomic nervous center that controls various functions of pelvic organs including micturition, defecation and penile erection (Fowler et al., 2008; de Groat et al., 2015). The parasympathetic preganglionic neurons (PPNs) of the SPN play a critical role in regulating these functions (Grill et al., 1999; Tai et al., 2004). Morphological studies have demonstrated that MOR-immunoreactive (IR) neurons are widely distributed in the spinal cord, including in the SPN (Gouardères et al., 1991; Mansour et al., 1994; Ding et al., 1996). The processes of PPNs are distributed extensively in the spinal cord, including in the dorsal commissural nucleus (DCN) in the superficial layers of the dorsal horn (Nadelhaft et al., 1980; Nadelhaft and Booth, 1984; Morgan et al., 1993), where they are immunopositive for MOR. Endomorphins, including endomorphin-1 (EM-1) and endomorphin-2 (EM-2), have high affinity and selectivity for MOR and are considered its endogenous ligands (Hackler et al., 1997; Zadina et al., 1997). Additionally, endogenous opioids have been suggested to play a critical role in the control of bladder function via the modulation of the parasympathetic outflow at the sacral cord level (Drenger et al., 1986). Our recent study revealed that EM-2-IR terminals form synapses with MOR-expressing PPNs in the rat SPN (Dou et al., 2013). To investigate the functional significance of these morphological results, we examined the effects of EM-2 on excitatory synaptic transmission and the neuronal excitability of PPNs in young rats (24–30 days old) using whole-cell patch-clamp recording. PPNs were identified by retrograde tracing with tetramethylrhodamine-dextran (TMR).

## Experimental Procedures

### Animals

Male Sprague Dawley (SD) rats aged 21–24 days were provided by the Experimental Animal Center of the Fourth Military Medical University (Xi’an, China). Rats were housed on a 12-h light–dark cycle (8 a.m. to 8 p.m. light) with access to food and water *ad libitum*. All animal experiments were approved by the Institutional Animal Care and Use Committee of the Fourth Military Medical University. All efforts were made to reduce the number of animals used and to minimize their suffering.

### Retrograde Labeling of PPNs

Parasympathetic preganglionic neurons (PPNs) in the sacral spinal cord were identified by retrograde labeling with TMR (3000 MW, Molecular Probe, Eugene, OR, USA). The procedure for the retrograde labeling operation was conducted according to that described in our previous works (Dou et al., 2013). For this purpose, the rats were anesthetized with a 2% sodium pentobarbital solution (40 mg/kg, *i.p*.), and the right pelvic nerve of each rat was exposed via a posterior approach through the sacrococcygeal region. After the dissection of the pelvic nerve, 1 μl of 10% TMR distilled in 0.1 M citrate-NaOH (pH 3; Kaneko et al., 1996) was applied to the surface of the nerve with a microsyringe.

### Slice Preparation

After the retrograde tracing operation, the rats were allowed to survive for 3–6 days. The rats were then anesthetized with 2% sodium pentobarbital (80 mg/kg, *i.p*.). After the disappearance of reflexes, the rats were perfused transcardially for 1 min with 100 ml of 2–4°C sucrose artificial cerebrospinal fluid (sucrose-aCSF) in which the NaCl had been replaced with sucrose (Mitra and Brownstone, 2012; Lu et al., 2013) and containing the following reagents (in mM): 220 sucrose, 2.5 KCl, 26 NaHCO_3_, 6.0 MgSO_4_, 1.2 KH_2_PO_4_, 0.5 CaCl_2_, 10 glucose, 1 ascorbate, and 3 sodium pyruvate. All aCSF solutions were bubbled with carbogen gas (95% O_2_ and 5% CO_2_). The spinal cord at L6-S1 was removed using pressurized sucrose-aCSF with a 2-ml syringe. Transverse slices (300 μm thick) were cut on a vibrating microtome (Leica VT 1200s, Heidelberger, Nussloch, Germany) in 2–4°C sucrose-aCSF bubbled with carbogen gas. The slices were transferred to an incubation chamber filled with normal-sodium aCSF (normal-aCSF) that was continuously bubbled with carbogen gas and incubated at room temperature (22–24°C). The normal-aCSF consisted of the following reagents (in mM): 124 NaCl, 2.5 KCl, 25 NaHCO_3_, 2 MgSO_4_, 1 NaH_2_PO_4_, 2 CaCl_2_, 10 glucose, 1 ascorbate, and 3 sodium pyruvate.

### Whole Cell Patch Clamp Recording

After a 1 h recovery period, the slices were transferred to recording chambers (volume 0.5 ml) mounted on a fixed-stage upright microscope (BX51W1, Olympus, Tokyo, Japan). The slices were continuously perfused with normal-aCSF bubbled with carbogen gas at a rate of 2–3 ml/min. The experiments were performed at 30 ± 1°C using a heat controller. The patch pipettes were constructed with a P-97 micropipette puller (Sutter Instruments, Novato, CA, USA) from borosilicate glass capillary tubes (World Precision Instruments, Sarasota, FL, USA). The tip resistances were in the range of 2–6 MΩ when filled with the pipette solution, which contained the following reagents (in mM): 130 potassium gluconate, 15 KCl, 5 NaCl, 10 4-(2-hydroxyethyl)-1-piperazineethanesulfonic acid (HEPES), 4 Mg-ATP, 0.3 Na_2_-GTP, and 0.4 ethylene glycol tetraacetic acid (EGTA). This solution was titrated to pH 7.3 with KOH. The osmolality of the pipette solutions was adjusted to 290–300 mOsm. To visualize the recorded neurons, 0.2% biocytin (Sigma-Aldrich, St. Louis, MO, USA) was added to the recording pipette solution. Whole cell patch-clamp recordings were performed on TMR-containing PPNs that were visualized under epifluorescence using a tetramethyl rhodamine isothiocyanate (TRITC) filter set (U-HGLGPS, Olympus) with a monochrome CCD camera (IR-1000E, DAGE-MTI, Michigan, USA) and monitor. The neurons were recorded using a Multiclamp 700B amplifier (Axon Instruments, Foster City, CA, USA). pCLAMP software (v. 10.02, Axon Instruments) was used to acquire and analyze the data. The signals were filtered at 2.6 kHz, digitized at 10 kHz (Digidata 1322A, Axon Instruments), and saved on a computer for offline analysis. Recordings that met the following criteria were included in the analyses: a resting membrane potential of at least −45 mV (the liquid junction potential was not corrected and was 13.7 mV in our experimental conditions), and a series resistance (Rs) was ≤30 MΩ. Average Rs did not change by more than 10% in any of the accepted recordings. A separate template was created for each recording by averaging a large number of hand-selected unambiguous spontaneous excitatory postsynaptic currents (sEPSCs) or miniature excitatory postsynaptic currents (mEPSCs).

### Immunohistochemical Staining for TMR, MOR and Biocytin

After recording, the slices were fixed in 4% paraformaldehyde in 0.1 M phosphate buffer (pH 7.4) for 4 h and were subsequently stored in 30% sucrose solution overnight at 4°C. After three rinses with 0.01 M PBS, the slices were processed for TMR, MOR and biocytin immunofluorescent staining. The sections were subjected to the following series of incubations. (1) The sections were incubated with a mixture of rabbit antiserum against TMR (1:200, A6397, Invitrogen, Eugene, Oregon, USA) and guinea pig antiserum against MOR (1:1000, GP10106, Neuromics, Edina, Minnesota, USA) in the antibody dilution medium for 72 h at 4°C. The medium consisted of 0.01 M PBS (pH 7.4) containing 5% (v/v) normal donkey serum (PBS-NDS), 0.3% (v/v) Triton X-100, 0.05% (w/v) NaN_3_ and 0.25% (w/v) carrageenan. (2) The sections were then incubated with a mixture of Alexa594-donkey anti-rabbit IgG (1:500, A21207, Invitrogen, Eugene, Oregon, USA) and Alexa647-goat anti-guinea pig IgG (1:500, A21450, Invitrogen, Eugene, Oregon, USA) in PBS-NDS for 4 h at room temperature. (3) Finally, the sections were incubated with fluorescein isothiocyanate (FITC)-labeled avidin (1:1000, A-2001, Vector, Burlingame, CA, USA) in PBS for 2 h at room temperature. After each step, the slices were washed three times with 0.01 M PBS. The slices were mounted, cover-slipped and examined using a confocal laser-scanning microscope (Olympus FV1000, Tokyo, Japan).

### Drugs and Chemicals

6-Cyano-7-nitroquinoxaline-2, 3-dione (CNQX), DL-2-amino-5-phosphonopentanoic acid (AP-5) and picrotoxin were purchased from Abcam (Cambridge, MA, USA). D-Phe-Cys-Tyr-D-Trp-Orn-Thr-Pen-Thr-NH2 (CTOP), 4-aminopyridine (4-AP), BaCl_2_ Endomorphin-2 (EM-2), guanosine 5′-[β-thio]diphosphate trilithium salt (GDP-β-S) and strychnine were purchased from Sigma-Aldrich (St. Louis, MO, USA). Tetrodotoxin (TTX) was purchased from Tocris (Tocris Bioscience, Bristol, UK). All drugs were prepared as stock solutions according to the manufacturer’s instructions and were stored frozen at −20°C. Before each experiment, the drug stock solutions were added to the normal-aCSF solution to obtain the experimental concentrations. The time required to completely exchange the solution in the recording chamber was 2 min. The drugs were applied by switching the bath chamber to another perfusion solution without changing the perfusion rate or temperature.

### Statistical Analysis

All numerical data are expressed as the means ± the SD. For the electrophysiology data, *n* refers to the number of neurons studied. The concentration–response relationships for multiple neurons were determined using Prism 5.0 (GraphPad, San Diego, CA, USA). Where appropriate, group means were compared using paired *t*-tests for paired data. Drugs were bath-applied for 8 min. The cumulative probabilities of the inter-event intervals and the amplitudes of the sEPSCs and mEPSCs were compared using Kolmogorov–Smirnov tests using the 3 min duration before drug application as the control amplitude or frequency for comparison with the amplitudes or frequencies of 3 min duration after 5 min of drug application. Differences between the means were considered significant at *P* < 0.05.

## Results

### Identification of the PPNs

**Figure 1:**
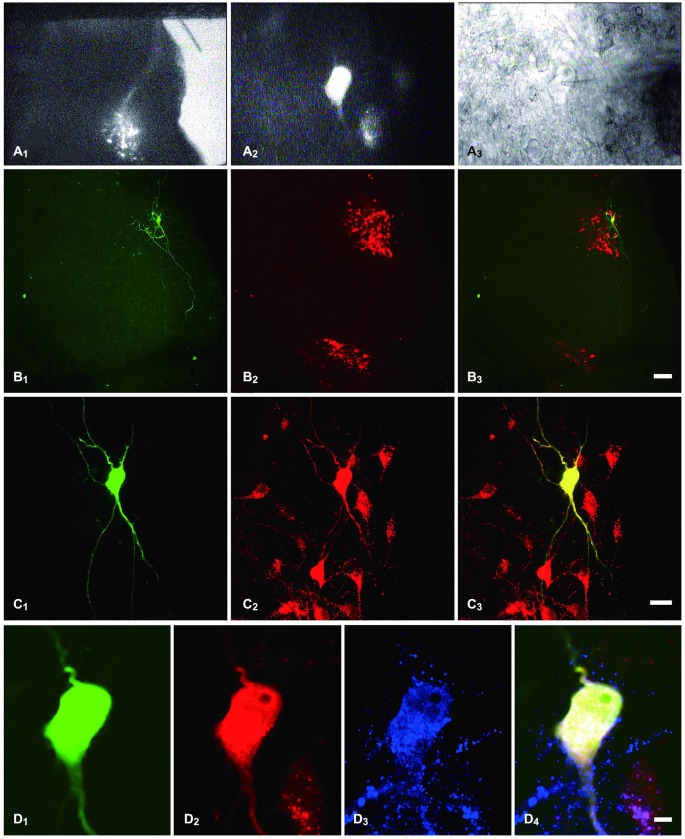
**Identification of PPNs and of the recorded PPNs that were immunostained for MOR. (A_1_)** sacral parasympathetic nucleus (SPN) located in the mediolateral border of the gray matter that was identified under a lower magnification with fluorescent illumination. **(A_2_)** tetramethylrhodamine-dextran (TMR)-labeled PPNs were identified at a higher magnification with fluorescent illumination. **(A_3_)** The neuron shown in **(A_2_)**, but viewed with infrared illumination during whole-cell recording. **(B_1_,C_1_,D_1_)** The recorded neuron shown in **(A_2_,A_3_)**, filled with biocytin and visualized with FITC-conjugate avidin (green) at various magnifications. **(B_2_,C_2_,D_2_)** The same section shows TMR immunoreactivity (Alexa 594) and is shown at the same magnification as in **(B_1_,C_1_,D_1_)**, respectively. **(B_3_,C_3_,D_4_)** Merged images show that the biocytin-filled neuron was the TMR-containing neuron. **(D_3_)** The neuron also shows MOR-Immunoreactive (IR) (Alexa 647). **(D_4_)** Merged image shows that the biocytin-filled neuron was the TMR-containing neuron that exhibited MOR-IR. Scale bars: 7 μm in **(B)**; 20 μm in **(C)**; 100 μm in **(D)**.

Consistent with previous reports (Nadelhaft and Booth, 1984), PPNs labeled with TMR were found in lumbosacral segments L6-S1 of the rat (Figure 1A_1_). We performed whole-cell patch-clamp recordings from PPNs that were retrogradely labeled with TMR (Figures 1A_1_,A_2_). Biocytin was introduced into the intracellular solution to visualize the recorded PPNs (Figures 1B_1_,C_1_,D_1_) which were labeled with TMR (Figures 1B_2_,B_3_,C_2_,C_3_,D_2_,D_4_). These results further confirm that the recorded neurons were PPNs.

### EM-2 Decreases both the Frequency and the Amplitude of sEPSCs and mEPSCs via MOR in PPNs

The spontaneous excitatory postsynaptic currents (sEPSCs) and miniature excitatory postsynaptic currents (mEPSCs) were recorded under whole-cell voltage clamping at −70 mV, and high-pass and low-pass filters were selected to eliminate the baseline fluctuations and noise. To examine the effects of EM-2 on the sEPSCs and mEPSCs of the PPNs, 100 μM picrotoxin and 2 μM strychnine were applied to eliminate the influence of inhibitory neurotransmission. At the end of the experiments, the sEPSCs and mEPSCs were blocked by bath application of the 10 μM α-amino-3-hydroxy-5-methyl-4-isoxazolepropionic acid (AMPA) receptor antagonist 6-cyano-7-nitroquinoxaline-2, 3-dione (CNQX), which confirmed that the sEPSCs and mEPSCs were glutamate-mediated postsynaptic current (*n* = 7 and *n* = 3, respectively, data not shown).

#### EM-2 Decreased the Amplitudes and Frequencies of sEPSCs

**Figure 2:**
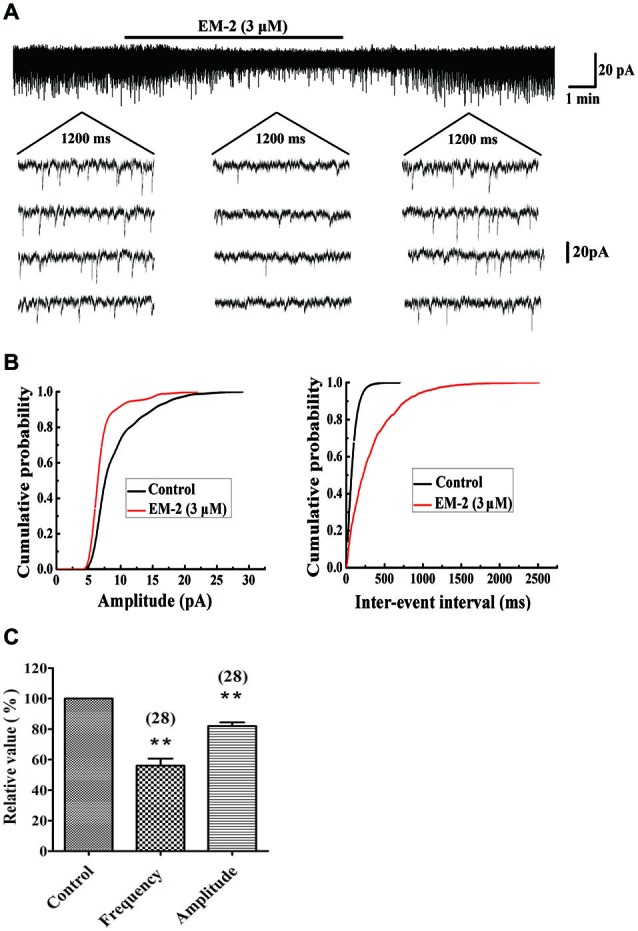
**EM-2 decreased amplitudes and frequencies of sEPSCs in PPNs. (A)** Sample traces of sEPSCs in the absence and presence of EM-2. In this and subsequent figures, the duration of drug superfusion is shown by a horizontal bar above the chart recording, and 4 consecutive traces of sEPSCs are shown in the expanded time scales. The period of each trace is 1200 ms. **(B)** Cumulative distributions of the amplitudes (left) and inter-event intervals (right) of the sEPSC before (black line) and during (red line) the EM-2 applications. EM-2 significantly shifted the mean cumulative distribution of the sEPSC amplitude to lower amplitudes (*P* < 0.01; Kolmogorov–Smirnov test) and significantly shifted the mean cumulative distribution of the sEPSC inter-event intervals to longer durations (*P* < 0.01; Kolmogorov–Smirnov test). The data were obtained from the same neuron as in panel **(A). (C)** Histogram showing that the sEPSC amplitude and frequency were significantly reduced by EM-2 (*P* < 0.01, paired *t*-test *n* = 28). In this and the following figures, the numbers of neurons used to construct each data point are shown in parentheses. The vertical bars show the SDs. **P < 0.01 vs. control. Holding potential = −70 mV.

The effects of EM-2 on sEPSCs were studied in 28 PPNs. In 23 of 28 tested PPNs, perfusion of EM-2 (3 μM) for 8 min resulted in a reversible reduction in amplitude of sEPSCs (Figures 2A,B). The remaining 5 PPNs showed a nonsignificant change in sEPSC amplitude. Overall sEPSC amplitude was reduced from –17.1 ± 3.9 pA in controls to –13.8 ± 3.2 pA with EM-2 (82 ± 12.5% of the control sEPSC amplitude, *P* < 0.01; paired *t*-test *n* = 28; Figure 2C). The sEPSC frequency was also significantly reduced in 25 of 28 tested PPNs (Figures 2A,B), increased in 1 of 28 tested PPNs, and unchanged in the remaining 2 PPNs. Overall, sEPSC frequency was reduced from 1.68 ± 0.85 Hz in the control to 0.87 ± 0.44 Hz with EM-2 (56 ± 24.5% of the control sEPSC frequency; *P* < 0.01; paired *t*-test, *n* = 28; Figure 2C).

#### CTOP Blocked the Action of EM-2 on sEPSCs

**Figure 3:**
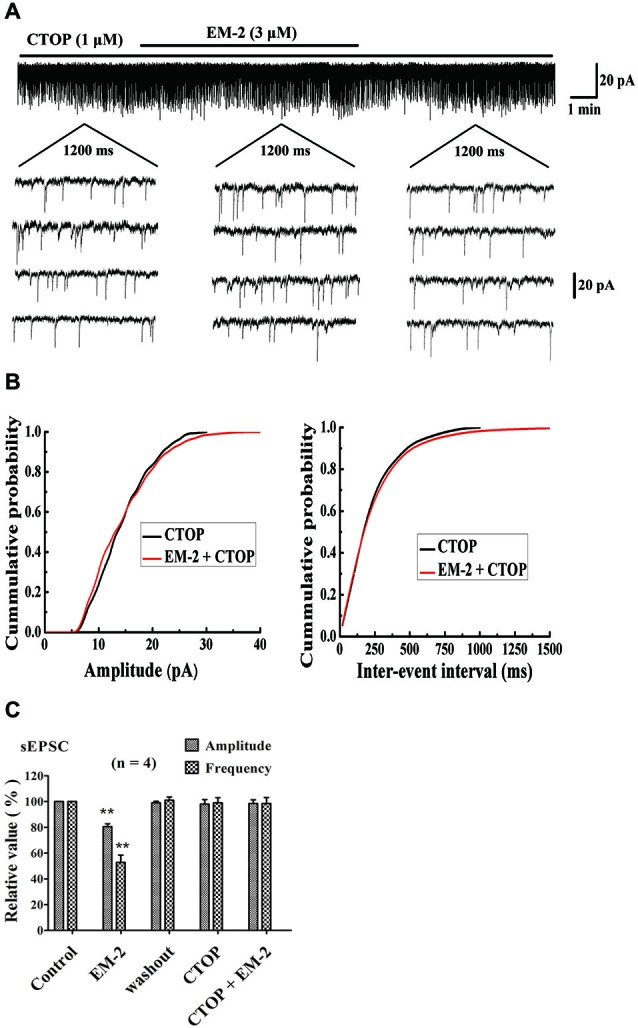
**Cys-Tyr-D-Trp-Orn-Thr-Pen-Thr-Nh2 (CTOP) blocked the action of EM-2 on sEPSCs in PPNs. (A)** A sample trace of sEPSCs in the absence and presence of EM-2 (3 μM) together with CTOP (1 μM). **(B)** Cumulative distributions of the amplitudes (left) and inter-event intervals (right) of the sEPSCs in the presence of CTOP (black line) and EM-2 together with CTOP (red line). EM-2 did not significantly shift the mean cumulative distribution of the sEPSC amplitude or the inter-event intervals (*P* > 0.05; Kolmogorov–Smirnov test). These data were obtained from the same neuron as in panel **(A). (C)** Bar graph show the analytical results of four continuous recordings during which different treatments were performed in four PPNs. EM-2 clearly decreased the amplitudes and frequency of sEPSCs in these 4 PPNs (*P* < 0.01). After the washout of EM-2, the amplitudes and frequency returned to control levels. CTOP had no effect on either the amplitudes or frequency of sEPSCs (*P* > 0.05). However, with perfusion of both CTOP and EM-2, EM-2 was unable to decrease the amplitudes or frequency of the sEPSCs (*P* > 0.05). **P < 0.01 vs. control. Holding potential = −70 mV.

Figures 3A,B show a recorded PPN that was sensitive to amplitude- and frequency-decreasing effects of EM-2, and these effects were abolished in the presence of the selective MOR antagonist D-Phe-CTOP (CTOP, 1 μM). We examined four PPNs in which the sEPSC amplitudes and frequencies were reduced by EM-2 (80.5 ± 2.3% of the control sEPSC amplitude and 52.8 ± 5.6% of the control sEPSC frequency, *P* < 0.01; paired *t*-test; *n* = 4; Figure 3C). After 10 min of washout of the EM-2, the amplitudes and frequencies returned to the control levels (99 ± 1.2% of the control sEPSC amplitude and 101 ± 2.5% of the control sEPSC frequency; *n* = 4; *P* > 0.05). Bath application of CTOP (1 μM) had no effect on the amplitudes or frequencies of the sEPSCs (98 ± 3.5% of the control sEPSC amplitude and 99 ± 4% of the control sEPSC frequency; *n* = 4; *P* > 0.05), and perfusion with EM-2 after pretreatment with CTOP had no effect on the sEPSC amplitudes and frequencies (98.5 ± 2.8% of the control sEPSC amplitude and 98.5 ± 4.6% of the control sEPSC frequency; *n* = 4; *P* > 0.05; Figure 3C).

#### Effect of TTX on the EPSCs

In the presence of the Na^+^-channel blocker tetrodotoxin (TTX, 1 μM), the recorded events at a voltage-clamped −70 mV were action potential-independent miniature EPSCs (mEPSCs). These responses are thought to reflect the quantal release of glutamate acting at non-NMDA receptors (Nicola and Malenka, 1997). In this study, the recorded EPSC absence of TTX named sEPSC, in the presence of TTX named mEPSC.

**Figure 4:**
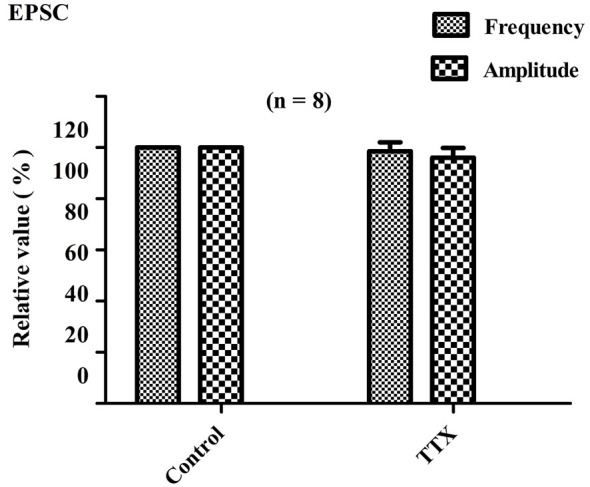
**Effects of TTX on EPSC frequency and amplitude**. Bar graph showing the effects of TTX (1 μM) on amplitudes and frequencies of EPSCs relative to control values. Perfusion with TTX did not significantly alter the average frequency and amplitude of EPSCs (*P* > 0.05; *n* = 8).

For all eight of the tested PPNs, the average amplitude and frequency of sEPSCs were −16.5 ± 6.3 pA and 2.01 ± 0.5 Hz, respectively. In eight of eight PPNs, the perfusion of TTX (1 μM) did not significantly alter the cumulative distribution of EPSC frequency (*P* > 0.05). The overall average EPSC frequency did not significantly change due to perfusion with TTX (98.5 ± 3.6% of the control sEPSCs frequency; *P* > 0.05; paired *t*-test, *n* = 8; Figure 4). In six of the eight PPNs, the amplitudes of the sEPSCs and the mEPSC were not significantly different (*p* > 0.05). In the two of eight PPNs that showed unusually large sEPSC amplitudes, the cumulative distribution of the EPSC amplitudes was significantly altered (*P* < 0.05; Kolmogorov–Smirnov test). However, the overall average EPSC amplitude did not significantly change due to perfusion with TTX (96 ± 3.8% of the control sEPSC amplitude; *P* > 0.05; *n* = 8; Figure 4). Comparison of the amplitudes between sEPSCs and mEPSCs revealed that TTX selectively eliminated the EPSCs with relatively large amplitudes, which were thought to be induced by action potential-dependent transmitter release.

#### EM-2 Decreased mEPSC Frequency and Amplitude

**Figure 5:**
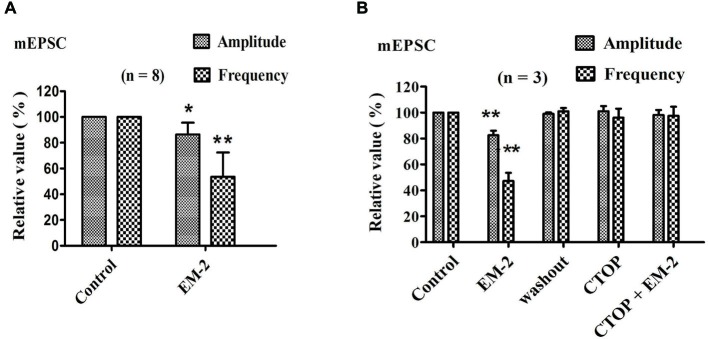
**EM-2 reduced the mEPSC amplitude and frequency via the activation of MORs. (A)** The effects of EM-2 on the amplitude and frequency of mEPSCs. Histogram showing that the mEPSC amplitude and frequency were significantly reduced by EM-2 (*P* < 0.05 and *P* < 0.01 respectively; *n* = 8). **(B)** Bar graph showing the analysis of three recordings during which different treatments were performed in three PPNs. EM-2 clearly decreased the amplitudes and frequency of sEPSCs in these 3 PPNs (*P* < 0.01). After the washout of EM-2, the amplitudes and frequency returned to control levels. CTOP had no effect on either the amplitudes or frequency of mEPSCs (*P* > 0.05). However, with perfusion of both CTOP and EM-2, EM-2 was unable to decrease the amplitudes or frequency of the sEPSCs (*P* > 0.05). *P < 0.05, **P < 0.01 vs. control.

The effects of EM-2 on mEPSCs were studied in eight PPNs. In five of eight tested PPNs, perfusion of EM-2 (3 μM) resulted in a reversible reduction in amplitude of mEPSCs (Figure 5A). The remaining three PPNs showed no significant changes in mEPSC amplitude. Overall mEPSC amplitude was reduced from –15.7 ± 5.2 pA in control to –13.5 ± 4.5 pA with EM-2 (86.4 ± 9.2% of the control mEPSC amplitude, *P* < 0.05; paired *t*-test *n* = 8; Figure 5A). The frequency of mEPSCs was also significantly reduced in seven of eight tested PPNs, with frequency being increased in the remaining one PPN. Overall mEPSC frequency was reduced from 1.98 ± 0.5 Hz in control to 1.09 ± 0.57 Hz in EM-2 (53.5 ± 18.9% of the control mEPSC frequency; *P* < 0.01; paired *t*-test, *n* = 8; Figure 5A).

#### CTOP Blocked the Action of EM-2 on mEPSCs

We further examined three PPNs in which the amplitude and frequency of mEPSCs were reduced by EM-2 (82.5 ± 3.6% and 47.2 ± 6.3% of the control mEPSC amplitude and frequency, respectively; *P* < 0.01; *n* = 3; Figure 5B). After 10 min of washout of EM-2, the amplitudes and frequencies returned to their control levels, and the bath application of CTOP (1 μM) had no effect on the amplitude or frequency of the mEPSCs (101 ± 4.1% of the control mEPSC amplitude and 96 ± 7% of the control mEPSC frequency; *P* > 0.05; *n* = 3). Similarly, perfusion with EM-2 in the pretreatment with CTOP had no effect on the mEPSC amplitudes and frequencies (98.2 ± 3.8% of the control mEPSC amplitude and 97.5 ± 7.1% of the control mEPSC frequency; *P* > 0.05; *n* = 3).

### EM-2 Decrease the Excitability of PPNs via Activation of MORs in the PPNs

#### EM-2 Hyperpolarized the Resting Membrane Potentials of the PPNs

**Figure 6:**
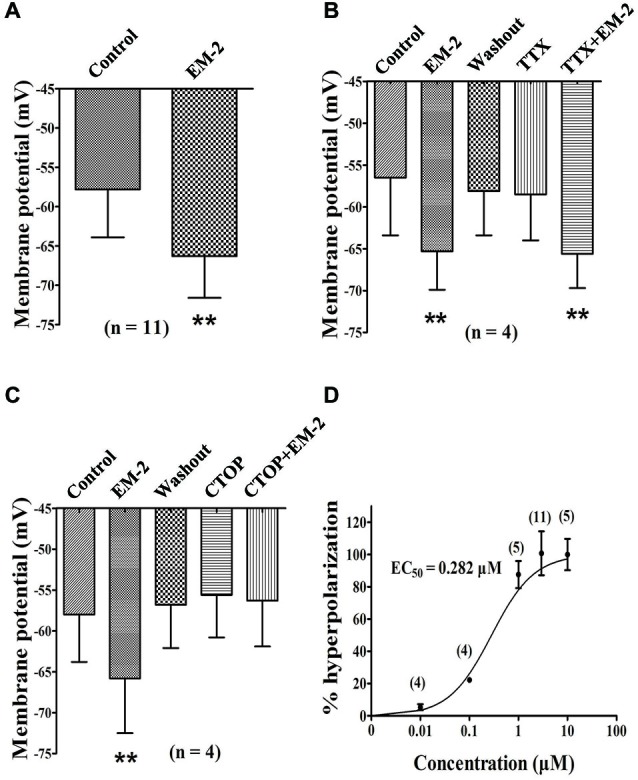
**EM-2 hyperpolarized the RMPs of the PPNs via the activation of MORs. (A)** Histogram showing that the average RMPs of the 11 responsive neurons decreased from −57.8 ± 6.1 mV to −66.3 ± 5.3 mV in the presence of EM-2 (*P* < 0.01, paired *t*-test *n* = 11). **(B)** The effect of TTX on RMPs. Bar graph showing the analysis of four recording during which different treatments were performed in these four PPNs. The RMPs of PPNs were hyperpolarized by EM-2 (*P* < 0.01). After the washout of EM-2, the RMPs of the recorded PPNs returned to the control levels. TTX alone had no effect on the membrane potential (*P* > 0.05; *n* = 4). TTX did not block the EM-2-induced membrane hyperpolarization (*P* < 0.01). **(C)** CTOP blocked the action of EM-2 on the RMPs. Bar graph showing the the analysis of four recording during which different treatments were performed in four PPNs. The RMPs were hyperpolarized by EM-2 (*P* < 0.01). After the washout of EM-2, the membrane potentials of the recorded PPNs returned to control levels. CTOP itself had no effect on membrane potential (*P* > 0.05). However, EM-2 did not produce membrane hyperpolarization in the presence of CTOP (*P* > 0.05). **(D)** Dose-response curve of the hyperpolarization of the PPNs by EM-2. The EC_50_ value was 0.282 μM. **P < 0.01.

These experiments were performed in current-clamp mode with zero holding current. In the presence of CNQX (10 μM), AP-5 (100 μM), picrotoxin (100 μM) and strychnine (2 μM) to eliminate the influences of excitatory and inhibitory transmission, the bath application of EM-2 (3 μM) produced a decrease in resting membrane potential in 11 of the 18 PPNs (61.1%). The average membrane potential of the 11 positively responding neurons decreased from −57.8 ± 6.1 mV to −66.3 ± 5.3 mV (*P* < 0.01; *n* = 11; Figure 6A) combined with a decrease in input resistance from 483.6 ± 226.5 MΩ to 365.6 ± 186.4 MΩ (*P* < 0.01; *n* = 11). Following the washout of EM-2, the membrane potential returned to its control level, and these 11 PPNs were further investigated. Four of these 11 PPNs were used to test the effects of TTX (1 μM) on membrane potentials. TTX alone had no effect on membrane potential (washout, −58.1 ± 5.3 mV compared with TTX, −58.5 ± 5.5 mV; *P* > 0.05; *n* = 4). With TTX + EM-2, the average membrane potential hyperpolarized to −65.6 ± 5.1 mV from −58.5 ± 5.5 mV with TTX alone (*P* < 0.01; *n* = 4 Figure 6B). These results indicate that TTX did not affect the EM-2 induced membrane hyperpolarization. Next, we examined the effect of CTOP on membrane potentials of another four PPNs. CTOP (1 μM) itself had no effect on membrane potential (washout, −56.8 ± 5.3 mV compared with CTOP, −55.6 ± 5.2 mV; *P* > 0.05; *n* = 4). However, EM-2 no longer produced membrane hyperpolarization in the presence of CTOP (CTOP, −55.6 ± 5.2 mV compared with EM-2 + CTOP, −56.3 ± 5.6 mV; *P* > 0.05; *n* = 4; Figure 6C).

#### Immunostaining for MOR in the above Recorded Neurons

At the end of the recording, the 18 slices described above were fixed for further examination of the MOR expression in the recorded PPNs. Eight of 11 PPNs in which membrane potentials were hyperpolarized by EM-2 exhibited MOR-IR (Figure 1D_3_). The seven PPNs whose membrane potentials were not responsive to EM-2 did not exhibit MOR-IR.

#### Dose-response Relationship Between EM-2 Concentration and Membrane Hyperpolarization

EM-2-induced membrane hyperpolarization tended to become more negative with increasing concentrations. Different concentrations of EM-2 were applied to assess the effect of a given concentration compared with the control. The dose-response curve for EM-2 is shown in Figure 6D, and the effective concentration that produced the half-maximal response (EC_50_) was 0.282 μM. EM-2 at 3 μM elicited the maximum effect, and further increases in the concentration did not increase the hyperpolarization. Therefore, 3 μM EM-2 was used in subsequent experiments in the present study.

#### EM-2 Induced Inwardly Rectifying K+ Currents through the G-Protein-Coupled Inwardly Rectifying Potassium (GIRK) Channel, and this Effect was Mediated by MORs

**Figure 7:**
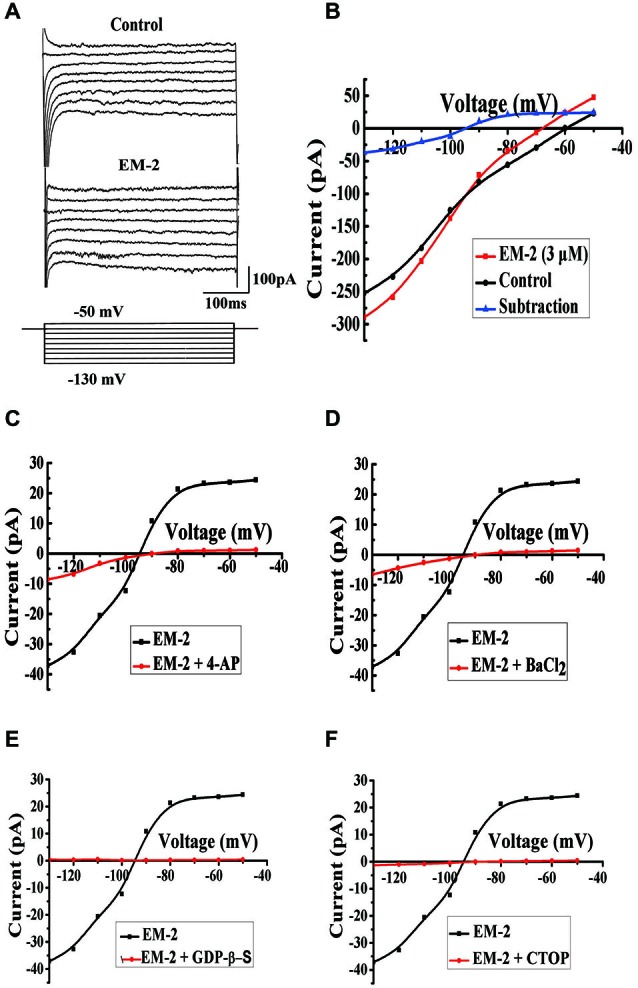
**Mechanism for EM-2-induced membrane current on PPNs. (A)** Current responses to voltage steps (bottom) in PPNs in the absence (top) and presence (middle) of EM-2. **(B)** The current amplitude, which was measured at the end of the voltage pulses, was plotted against voltages in the absence (black) and presence (red) of EM-2. The net EM-2-induced current (blue) was subtracted from the difference between the current responses in the absence and presence of the EM-2-induced current. **(C,D)** The average EM-2-induced current was significantly decreased by perfusion with 4-AP (1 mM, *n* = 5) or BaCl_2_ (1 mM, *n* = 4). **(E)** The average EM-2-induced current was blocked by the addition of GDP-β-S (2 mM) to the pipette solution following adequate exchange of the pipette solution for 15–20 min (*n* = 4). **(F)** Perfusion with CTOP (1 μM) blocked the average current activation by EM-2 (*n* = 4).

To examine the types of channels that were involved in the EM-2-induced membrane hyperpolarization, we compared currents induced by step voltage pulses in the absence and presence of EM-2 (Figure 7A). Eight step voltage pulses of 400 ms per step were applied, ranging from −130 to −50 mV, from the holding potential of −60 mV. In these experiments the aCSF solution contained TTX (1 μM), CNQX (10 μM), AP-5 (100 μM), picrotoxin (100 μM) and strychnine (2 μM). Figure 7B shows that the step voltage pulses induced relative currents in the absence (black) and presence (red) of EM-2. The net EM-2-induced current (blue), which was derived from the difference between the two currents, exhibited a clear reversal and inward rectification. The measured reversal potential was −95 ± 2.5 mV (*n* = 6), which is close to the value of the equilibrium potential of K^+^ (−107 mV) as calculated with the Nernst equation for our experimental conditions in which the pipette and aCSF K^+^ concentrations were 145 mM and 2.5 mM, respectively. This slight difference might reflect a liquid junction potential (13.7 mV) between the aCSF and pipette solutions. The EM-2-induced currents were significantly decreased in the presence of the potassium channel blocker 4-aminopyridine (4-AP, 1 mM) (Figure 7C, *n* = 5) or BaCl_2_ (1 mM; Figure 7D, *n* = 4). These results suggest that EM-2 activated an inwardly rectifying K^+^ current. After the application of the GDP analog guanosine 5′-[β-thio]diphosphate trilithium salt (GDP-β-S, 2 mM) to the recording pipette (15–20 min after rupture), EM-2 (3 μM) evoked no obvious net current (Figure 7E, *n* = 4), suggesting that this current involved G-protein-coupled channels. Additionally, no obvious current was activated by EM-2 (3 μM) in the presence of CTOP (1 μM; Figure 7F, *n* = 4), suggesting that the channel activation induced by EM-2 was mediated by MORs. Taken together, these results suggest that EM-2 induced inwardly rectifying K^+^ currents through the G-protein-coupled inwardly rectifying potassium (GIRK) channel and that this effect was mediated by MORs.

#### EM-2 Increased the Rheobase Current and Reduced the Repetitive Firing of PPNs

**Figure 8:**
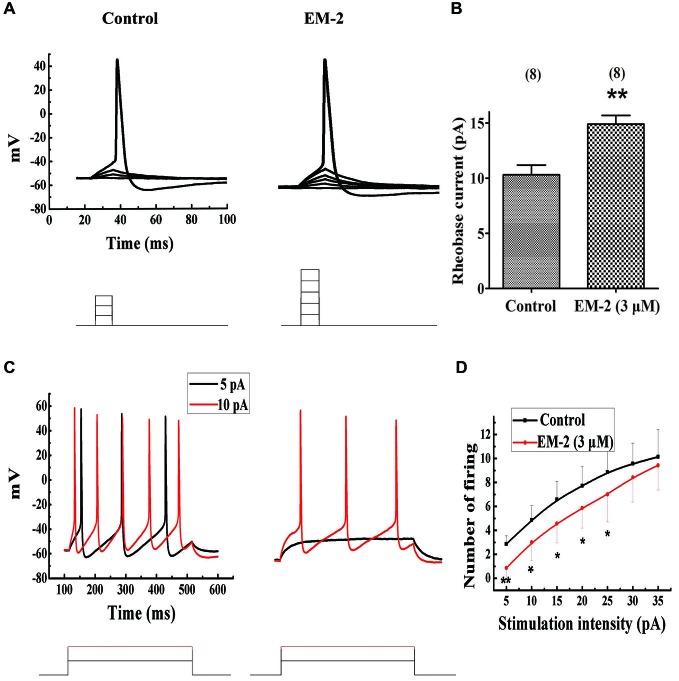
**EM-2 increased the rheobase current and inhibited the repetitive firing of PPNs. (A)** A sample traces showing EM-2 (3 μM) increased the rheobase current (from 6 pA to 10 pA) and hyperpolarized the RMPs from −52.8 mV to −60.2 mV (step = 2 pA and duration = 15 ms). **(B)** EM-2 (3 μM) significantly increased the rheobase current from a mean of 10.3 ± 2.5 pA to 14.9 ± 2.2 pA (*P* < 0.01; *n* = 8). **(C)** A sample trace showing that EM-2 (3 μM) decreased the numbers of spikes in the PPNs in response to progressively depolarizing current injections with a step size of 5 pA and a 400 ms duration (bottom). EM-2 hyperpolarized the neuron from −58.2 mV to −64.2 mV. **(D)** The relationship between the numbers of spikes in PPNs and the amounts of depolarizing current injected into the neurons. EM-2 reduced repetitive action potential firing over the entire range of injected currents (5 pA-25 pA) (*P* < 0.05). However, EM-2 (3 μM) did not significantly reduce the number of action potentials evoked by larger injected currents (30 pA-35 pA) (*P* > 0.05). **P < 0.01, *P < 0.05.

The aCSF with 10 μM CNQX, 100 μM AP-5, 100 μM picrotoxin and 2 μM strychnine was used to eliminate the influence of excitatory and inhibitory transmission. Using current-clamp mode and pulse steps of depolarizing current (steps of 1 pA or 2 pA for 15 ms, until action potentials were induced), EM-2 (3 μM) significantly increased the rheobase current (the minimum current required to elicit an action potential; Figure 8A) in 8 of 15 tested PPNs, from a mean of 10.3 ± 2.5 pA to 14.9 ± 2.2 pA (a mean change of 4.6 ± 1.2 pA; *P* < 0.01; *n* = 8; Figure 8B). The above 8 PPNs were further examined to determine the effects of EM-2 on the repetitive firing evoked by the application of progressively more depolarizing current injection with steps of 5 pA for 400 ms durations in the current-clamp mode. Figure 8C shows an example of the effects of EM-2 (3 μM), which decreased the number of action potentials of PPNs in response to progressively depolarizing current injections. Meanwhile, EM-2 (3 μM) hyperpolarized the neuron from −58.2 mV to −64.2 mV. Figure 8D shows that EM-2 reduced repetitive action potential firing at multiple levels of injected current (5 pA-25 pA, *P* < 0.05; *n* = 8, Figure 8D). This increase in rheobase current and reduction in repetitive firing was due to a decrease in the RMPs (from −57.8 ± 6.1 mV to −66.3 ± 5.3 mV; *P* < 0.05; *n = 8)*.

## Discussion

Previous studies revealed that Endomorphin-1 (EM-1) is widely and densely distributed throughout the brain and upper brainstem (Martin-Schild et al., 1999). However, endomorphin-2 (EM-2)-IR varicose fibers were mainly observed in the substantia gelatinosa of the medulla and the spinal cord dorsal horn (Martin-Schild et al., 1999; Pierce and Wessendorf, 2000). Additionally, our recent study revealed that EM-2-IR terminals form synapses with MOR-expressing PPNs in the rat SPN (Dou et al., 2013). Therefore, EM-2 was selected for the examination of the effects of opioids on PPNs in the present study. Previous studies of the spinal substantia gelatinosa (Yoshimura and North, 1983; Schneider et al., 1998), trigeminal substantia gelatinosa (Grudt and Williams, 1994), spinal ventral horn (Honda et al., 2012) and locus coeruleus (Williams et al., 1982) have shown that μ-opioid agonists hyperpolarize the membrane potentials of these neurons via the activation of K^+^ channels. Further, previous studies have demonstrated that MOR agonists such as morphine are involved in the inhibition of bladder control (Dray and Metsch, 1984a,b) and the inhibition of the micturition reflex at the level of the spinal cord in rat (Hisamitsu and de Groat, 1984). However, patch-clamp recordings examining μ-opioid modulation of PPNs have not been reported, and little is known regarding the effects of MOR activation in PPNs. Therefore, in this study, we examined the cellular effects of EM-2 on PPNs of the rat SPN. We demonstrated that EM-2 inhibited the excitatory neurotransmitter release from presynaptic terminals onto PPNs and hyperpolarized the RMPs of the PPNs, resulting in decreases in the excitability of the PPNs. We have observed the similar effects of EM-1 on the PPNs. Merely, the distribution of EM-1 is less than EM-2 in the spinal cord, and the physiological significant of EM-1 to the bladder function is less important than EM-2, so we focused on the effects of EM-2 on PPNs in the present study. The results of the present study could not exclude the affection of EM-1 on bladder functions, and what is the effect of EM-1 on bladder function need to be further investigated in the future.

### EM-2 Decreased both the Frequency and the Amplitude of the PPN sEPSCs and mEPSCs via MORs

Measurements of the changes in random quantal transmitter release events are a sensitive means to estimate the locus of a drug’s effect. Typically, changes in mEPSC amplitudes are associated with a postsynaptic site of drug action, whereas changes in mEPSC frequency are likely due to an interaction with a presynaptic site that changes the probability of quantal transmitter release (Lupica, 1995). Previous studies have demonstrated that DAMGO and EM-2 act both pre- and postsynaptically to alter glutamatergic transmission in the substantia gelatinosa (Fujita and Kumamoto, 2006) and the spinal ventral horn (Honda et al., 2012).

Using the application of both picrotoxin and strychnine to eliminate the influence of inhibitory transmission, the present results revealed that the spontaneous excitatory postsynaptic currents (sEPSCs) were completely blocked by the AMPAR antagonist CNQX, indicating that these sEPSCs were mediated by glutamatergic AMPA receptors, as reported previously (Yoshimura and Nishi, 1993). Recordings of glutamatergic sEPSCs may consist of a mixture of presynaptic action potential-dependent transmitter release and random quantal transmitter release (Scanziani et al., 1992). However, in this study, their frequency and amplitudes were independent of sodium channels, as shown by blockade with 1 μm tetrodotoxin (TTX), suggesting that most of the sEPSCs represented genuine mEPSCs. These results are consistent with those of previous studies of substantia gelatinosa neurons (Fujita and Kumamoto, 2006; Jiang et al., 2014) and ventral horn neurons (Honda et al., 2012) in rat spinal cord slices.

The EM-2-induced decrease in the amplitudes of mEPSCs was blocked by the application of the selective MOR antagonist CTOP. These results suggest that the EM-2-induced inhibitory effect on PPNs was mediated by the activation of postsynaptic MORs. The EM-2-evoked membrane potential hyperpolarization might account for the reductions in the amplitudes of sEPSCs and mEPSCs. Further discussion of EM-2-induced membrane hyperpolarization of the PPNs is presented below.

EM-2 also decreased the frequency of mEPSCs, and these effects were blocked by the application of CTOP. These results suggest that MORs were expressed on the presynaptic terminals and that the activation of these MORs decreased glutamate neurotransmitter release from the presynaptic terminal onto the PPNs. There is no direct morphological evidence to account for this functional phenomenon. However, indirect evidence from our previous studies has demonstrated that MORs are located in primary afferent neurons containing substance P (SP) or calcitonin gene-related peptide (Li et al., 1998). Additionally, our recent study revealed that EM-2, SP and MOR coexist within the dorsal root ganglion, spinal dorsal horn and primary afferent fibers and terminals. Moreover, it has been shown that PPNs send dendrites into the dorsal commissure and into the lateral funiculus and lateral edge dorsal horn of the spinal cord (Nadelhaft et al., 1980; Nadelhaft and Booth, 1984; Morgan et al., 1993), and these regions show heavy immunostaining for MORs (Ding et al., 1996). These morphological characteristics suggest that PPNs might form synaptic connections with terminals or somata of neurons that express MORs and that EM-2 may interact with presynaptic MORs, resulting in inhibition of excitatory neurotransmitter release from presynaptic terminals onto postsynaptic membrane of PPNs (Kohno et al., 1999; Fujita and Kumamoto, 2006).

The cellular mechanism underlying the presynaptic effects of EM-2 on PPNs was not examined in this study. Because previous studies have demonstrated that endomorphins inhibit high-threshold Ca^2+^ channel currents in rodent NG 108–15 cells overexpressing MOR (Higashida et al., 1998), we speculate that these effects might be due to an inhibition of the voltage-gated Ca^2+^ channels in nerve terminals. Whether EM-2 affects GABA or glycine neurotransmission onto PPNs requires further investigation.

In the present study, we observed a few recorded neurons that exhibited enhancements in EPSCs in response to perfusion of EM-2. Although we had added picrotoxin and strychnine to block the inhibitory neurotransmission mediated by GABA_A_ receptors and glycine receptors, because we did not use a GABA_B_ receptor antagonist, this response may be mediated by GABA_B_ receptors. It has been reported both GABA_A_ and GABA_B_ receptors are responsible for regulating the activity of PPNs (Nanninga et al., 1989; Araki, 1994). Presynaptic disinhibition of inhibitory interneurons may account for this phenomenon, as reported previously (Vaughan et al., 1997; Morgan and Clayton, 2005; Lau and Vaughan, 2014).

### EM-2 Hyperpolarized Resting Membrane Potential of PPNs via Activation of MOR

Previous studies of the spinal substantia gelatinosa (Yoshimura and North, 1983; Schneider et al., 1998), trigeminal substantia gelatinosa (Grudt and Williams, 1994), spinal ventral horn (Honda et al., 2012) and locus coeruleus (Williams et al., 1982) have shown that μ-opioid agonists exert their inhibitory effects via hyperpolarization of postsynaptic neurons that are sensitive to the MOR antagonist. Consistent with the above-mentioned inhibitory effects of opioids in other regions of the CNS, the present study demonstrated that EM-2 directly inhibited the PPNs. Bath application of EM-2 produced membrane hyperpolarization in 61.1% of the recorded PPNs. This percentage is similar to that observed in patch-clamp studies of spinal lamina IX neurons (Honda et al., 2012). The EC_50_ value of 0.282 μM for the activation of MORs on the postsynaptic membranes of the PPNs is similar to that reported previously (Fujita and Kumamoto, 2006). EM-2 significantly hyperpolarized PPNs in the presence of a mixture of the blocking agents CNQX, AP-5, picrotoxin and strychnine, which was applied to eliminate the influences of excitatory and inhibitory transmission. Additionally, EM-2-induced membrane hyperpolarization was not affected by the presence of TTX. These results indicate a direct effect of EM-2 on PPNs. Moreover, the EM-2-induced membrane hyperpolarization was antagonized by the selective MOR antagonist CTOP. Furthermore, 8 of the 11 recorded PPNs in which the membrane potentials were hyperpolarized by EM-2 exhibited MOR-IR. These results confirm that EM-2 hyperpolarized the membrane potentials of the PPNs via the activation of MORs on the PPNs. The remaining 3 PPNs were not IR for MOR, which may reflect the sensitivity of the antibody. These postsynaptic effects of EM-2 on PPNs are consistent with previous immunohistochemical (Ding et al., 1996; Moriwaki et al., 1996), autoradiographic (Gouardères et al., 1991) and *in situ* hybridization histochemical studies (Mansour et al., 1994) reporting that MORs are distributed in the SPN of rat spinal cord. These results further support our recent morphological evidence that EM-2-IR terminals form synapses with the MOR-expressing PPNs in the SPN of the rat L6-S1 lumbosacral spinal cord (Dou et al., 2013).

### Mechanism of EM-2 Induced Membrane Hyperpolarization on PPNs

In other regions of the CNS, the selective MOR agonist DAMGO has been reported to activate GIRK channels (Schneider et al., 1998; Honda et al., 2012). To explore the mechanism by which EM-2 induced membrane hyperpolarization in the PPNs in the L6-S1 lumbosacral spinal cord of the rat, we further examined whether EM-2 also activated GIRK channels. Our data demonstrate that EM-2-induced currents were reversed at a potential that was close to the predicted potassium equilibrium potential, exhibited a clear reversal and were inwardly rectifying. Additionally, the EM-2-induced currents were largely blocked by presence of the potassium channel blockers BaCl_2_ or 4-AP in aCSF. These results indicate that EM-2 activated inwardly rectifying potassium channels. Moreover, the average EM-2-induced current was blocked by the addition of GDP-β-S to the pipette solution. These results suggest that the inhibition of PPNs is dependent on the activation of G-proteins. The present results indicate that EM-2 activated GIRK channels. Furthermore, no current was activated by EM-2 in the presence of CTOP, suggesting that the channel activation by EM-2 was mediated by MORs. Taken together, these results suggest that the direct inhibitory postsynaptic effect of EM-2 on PPNs was mediated by a direct mechanism involving MOR activation coupled to GIRK channels and the subsequent hyperpolarization of the membrane potential.

### EM-2 Decreased the Excitability of PPNs

EM-2 significantly increased the minimum current required to elicit an action potential and reduced the repetitive firing of action potentials, as we reported recently of the similar effect of EM-1 in the rat ventrolateral periaqueductal gray (Chen et al., 2015). These effects of EM-2 on the PPNs were combined with a decrease in the membrane potentials of the PPNs. Additionally, the threshold for action potential generation was not significantly altered by the application of EM-2. These results indicate that the increase in rheobase current and the reduction of repetitive firing of the PPNs were due to hyperpolarization of the PPNs: such hyperpolarization moved the membrane potential further from the threshold for action potential generation. These results suggest that EM-2 directly decreased the excitability of the PPNs via the hyperpolarization of the membrane potentials of PPNs, which in turn resulted in an increase in the rheobase currents of PPNs and affected PPN function.

Micturition is a complex behavior involving cortical (Nadelhaft and Vera, 1995, 2001; Griebling, 2012; Nishijima et al., 2012), subcortical (Blok, 2002), brainstem (de Groat, 2002; Nishijima et al., 2005; Sugaya et al., 2005), spinal cord (Beckel and Holstege, 2011a,b) and bladder mechanisms (Birder et al., 2010; Wang et al., 2010; Birder and Wyndaele, 2013; Birder, 2013). In the spinal cord, neurons involved in the regulation of micturition are located in the superficial dorsal horn (Buss and Shefchyk, 2003), the dorsal gray commissure (Ding et al., 1997), Onuf’s nucleus (Mannen, 2000) and the SPN (De Groat et al., 1982). The PPNs in the SPN play a critical role in the processes of the micturition reflex (De Groat, 1975). In the intact spinal cord of adult animals, micturition is fulfilled mainly by multisynaptic reflexes that consist of micturition-related nuclei at the supraspinal cord level (Fowler et al., 2008; Nambiar and Lucas, 2014; de Groat et al., 2015). However, in chronically spinalized animals after spinal cord injury at cervical or thoracic levels, the primary sensory afferents make direct synaptic connections with the processes of the PPNs (Yoshimura et al., 2014; de Groat et al., 2015). Our previous studies using capsaicin treatment or rhizotomy to disrupt the normal transportation of EM-2 in primary afferents indicated that the major source of EM-2-IR fibers and terminals in the spinal cord is the ipsilateral primary afferent fibers (Hui et al., 2010; Zhu et al., 2011). Additionally, previous studies have demonstrated that numerous primary afferents from the bladder project to the SPN (Pascual et al., 1993). Moreover, our recent study has revealed that EM-2-IR terminals form synapses with MOR-expressing PPNs in the rat SPN (Dou et al., 2013). Based on these results, we concluuded that the primary sensory afferents from the bladder are important contributors of EM-2-IR terminals onto PPNs in the SPN region (Dou et al., 2013).

A previous study using cystometry in the bladder has shown that activation of MORs reduces the contraction of the detrusor (Dmitrieva and Berkley, 2002). Additionally, previous studies have provided compelling evidence that in the rat spinal cord, inhibition of micturition is mediated by MORs (Hisamitsu and de Groat, 1984; Kontani and Kawabata, 1988). Moreover, previous studies in an isovolumetric rat model have demonstrated that bladder contractions may be inhibited by the exogenous MOR agonist morphine, and this effect is abolished by the intravenous administration of the MOR antagonist naloxone (Dray and Metsch, 1984a). These results provide further evidence that the EM-2 induced decrease in the excitability of PPNs leads to inhibition of the excitatory information outflow from PPNs to parasympathetic postganglionic neurons.

The results of the present study reveal that EM-2 activated pre- and post-synaptic MORs, inhibiting excitatory neurotransmitter release from the presynaptic terminals and decreasing the excitability of PPNs due to hyperpolarization of their membrane potentials, respectively. Based on the results of the previous work and our present study, we speculate that information regarding bladder distention travels via afferent fibers within the pelvic nerve into the lumbosacral spinal cord. In the SPN, these EM-2-IR fibers form synaptic connections with MOR-IR PPNs. EM-2 is released from the presynaptic bouton and binds with MORs in the postsynaptic membrane, resulting in decreased excitability of PPNs due to hyperpolarization of their membrane potentials. The released EM-2 may also bind with presynaptic MORs, resulting in inhibiting excitatory neurotransmitter release from the presynaptic terminals. Both of these inhibitory effects of EM-2 on PPNs are mediated by MOR via pre- and/or post-synaptic mechanisms and contributed to the reduction of the excitatory information transmitted via the PPNs to the parasympathetic postganglionic neurons, resulting in a significant attenuation in the contractions of the rat bladder. Finally, these effects influence the micturition reflex formed by the synaptic connections between primary afferent terminals and PPNs, resulting in urinary retention.

## Conclusion

The present results indicate that the inhibitory effects of EM-2 on PPNs are mediated by MORs via pre- and/or postsynaptic mechanisms and affect PPNs-related functions. On the one hand, activation of presynaptic MORs by EM-2 caused the inhibition of excitatory neurotransmitter release from presynaptic terminals, on the other hand, EM-2 activated postsynaptic MORs and subsequently hyperpolarized the RMPs of the PPNs, resulting in decreases in the excitability of the PPNs. Both of these inhibitory effects of EM-2 contribute to the inhibition of micturition reflex formed by the synaptic connections between primary afferent terminals and PPNs. These inhibitory effects of EM-2 on PPNs at the spinal cord level may explain the mechanism of action of morphine treatment and morphine-induced bladder dysfunction in the clinic.

## Conflict of Interest Statement

The authors declare that the research was conducted in the absence of any commercial or financial relationships that could be construed as a potential conflict of interest.
